# A review on the beneficial and detrimental roles of gut microbiota-derived trimethylamine-N-oxide (TMAO)

**DOI:** 10.1016/j.imj.2026.100290

**Published:** 2026-10-10

**Authors:** Erta Rajabi, Zahra Pourramezan Seyedi, Mahsa Ghamari, Roghayeh Mohammadzadeh, Mehdi Mansouri, Friedrich Götz, Narjess Bostanghadiri

**Affiliations:** a Faculty of Medicine, Tehran University of Medical Sciences, Tehran, Iran; b Department of Biology, Faculty of Basic Sciences, Science and Research Branch, Islamic Azad University, Tehran, Iran; c Department of Pathobiology, School of Public Health, Tehran University of Medical Sciences, Tehran, Iran; d Food Safety Research Center (salt), Semnan University of Medical Sciences, Semnan, Iran; e Department of Pathobiology, Ka.C., Islamic Azad University, Karaj, Iran; f Department of Microbial Genetics, Interfaculty Institute of Microbiology and Infection Medicine Tübingen, University of Tübingen, Tübingen, Germany; g Research Center for Clinical Virology, Tehran University of Medical Sciences, Tehran, Iran

**Keywords:** Trimethylamine N-Oxide, Disorder, Microbiome

## Abstract

In recent years, increasing attention has focused on the role of the gut microbiome in converting dietary substances into bioactive metabolites implicated in the etiology of various diseases. Among these metabolites, trimethylamine-N-oxide (TMAO), a gut microbial metabolite derived from choline, carnitine, and betaine, has emerged as a novel molecule of interest as an alternative messenger with multiple, tissue-specific effects on human physiology. Once considered a benign osmolyte in marine organisms, TMAO is now implicated in human health risks through mechanisms involving oxidative stress, inflammation, and metabolic regulation. While elevated TMAO levels are associated with cardiovascular disease, chronic kidney disease, and neurodegeneration, evidence also highlights its cytoprotective properties, including protein stabilization and osmoprotection. This review provides a comprehensive analysis of TMAO's dual role in oxidative stress, inflammation, and endothelial mechanisms, clarifying its context-dependent effects and therapeutic implications.

## Highlights

• TMAO can promote ROS generation and inflammation or support antioxidant defenses via Nrf2 signaling.
• Elevated TMAO is associated with cardiovascular, neurodegenerative, metabolic, and kidney diseases.
• TMAO effects depend on microbiome composition, dietary intake, and disease context.

## Graphical abstract

**Figure:**
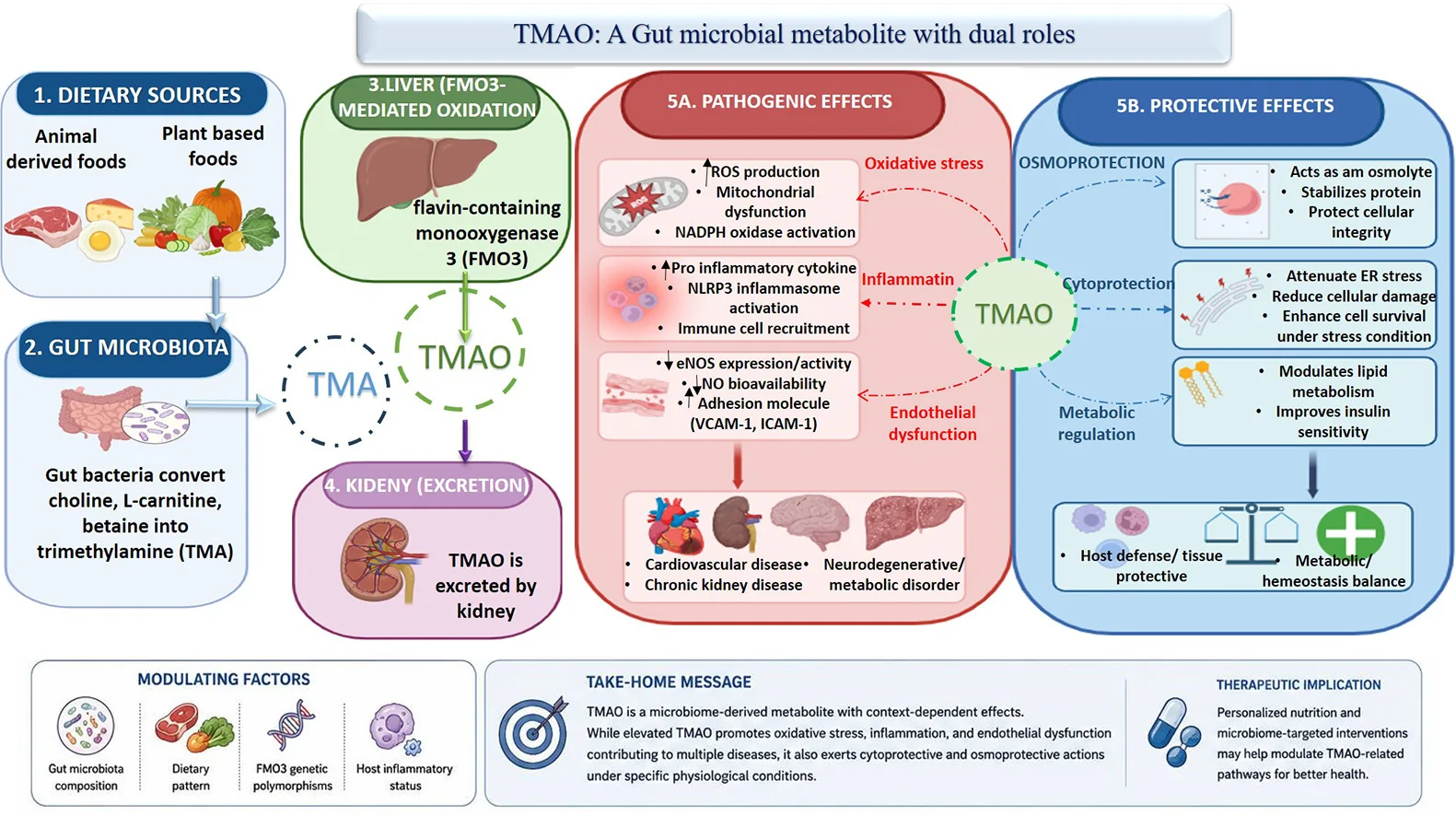

## 1 Introduction

The human gut microbiota is a densely populated, metabolically active ecosystem comprising trillions of microorganisms in the gastrointestinal tract. While bacteria dominate, viruses, fungi, and archaea also contribute to its diversity. Through intricate microbial interactions and continuous communication with host tissues, the gut microbiota profoundly affects host physiology and homeostasis. In recent years, increasing attention has focused on the interaction between gut microbiota and host metabolism, particularly how microbial metabolites derived from dietary components influence human health and disease.^1^ Gut microbes metabolize dietary substrates to produce a wide range of bioactive compounds, including short-chain fatty acids, secondary bile acids, and trimethylamine N-oxide (TMAO).^2^ TMAO is generated from dietary trimethylamine precursors, which are abundant in animal-derived foods such as red meat, fish, and egg yolk.^3^ TMAO formation occurs in two steps: gut bacteria convert dietary nutrients such as choline, L-carnitine, and betaine into trimethylamine (TMA). The liver then metabolizes TMA via flavin-containing monooxygenase (FMO) 3 to form TMAO. Factors such as gut microbiota composition, diet, FMO3 genetic polymorphisms, and host inflammatory status influence this conversion.^4^ In a screening study by Romano et al.^5^, eight bacterial species representing two different phyla (Firmicutes and Proteobacteria) and six genera were identified from the American Type Culture Collection and the German Collection of Microorganisms and Cell Cultures collections as TMA producers, all of which showed significant choline consumption and TMA accumulation: *Anaerococcus hydrogenalis, Clostridium asparagiforme, Clostridium hathewayi, Clostridium sporogenes, Edwardsiella tarda, Escherichia fergusonii, Proteus penneri*, and *Providencia rettgeri.*^5^ TMAO exerts context-dependent effects on metabolic tissues. Elevated TMAO is linked to adverse outcomes in cardiovascular, liver, and kidney diseases. However, under certain conditions, it can play a protective role by modulating oxidative and endoplasmic reticulum stress. Its effects vary across tissues and disease states, influencing lipid metabolism, insulin sensitivity, and cellular homeostasis. Overall, TMAO is a complex metabolite with both potential benefits and harms, warranting further mechanistic investigation.^6^ This study aims to provide a comprehensive overview of TMAO’s dual roles, focusing on its contribution to disease pathology. By examining the molecular mechanisms underlying both pathogenic and cytoprotective effects, we aim to inform the development of targeted therapies to modulate TMAO-related pathways in clinical practice.

## 2 Gut-liver-kidney axis signaling

The production of TMAO entails a two-step gut-liver axis process and the interaction between the host and intestinal microbiota; dietary precursors such as the metabolites including, L-carnitine, phosphatidylcholine, and betaine are initially metabolized into TMA, commonly found in animal-based foods including red meat, eggs, and high-fat dairy products.^7^ Conversely, vegetarian and Mediterranean diets correlate with markedly reduced TMAO concentrations, as one study demonstrated that an 8-week vegan diet decreased mean TMAO levels from 10.7 µmol/L at baseline to 5.66 µmol/L at week 1 and 6.38 µmol/L at week 8 in adults with dysglycemia; Moreover, a Mediterranean-style eating pattern with moderate unprocessed lean red meat consumption (200 g/week) reduced fasting serum TMAO in overweight/obese adults, whereas a higher red meat intake (500 g/week) within the same dietary pattern did not produce this reduction and resulted in significantly higher post-intervention TMAO concentrations.^8^^,^^9^ It is clinically important to note that current evidence does not support complete elimination of TMAO-precursor-rich foods; rather, a balanced approach focused on reducing processed and red meat consumption while increasing dietary fiber intake appears more appropriate, particularly for individuals with underlying cardiovascular or renal disease, as personalized, microbiome-directed dietary approaches warrant continued investigation to optimize TMAO reduction while preserving the essential physiological roles of choline and carnitine.^10^

The gut microbiome is the primary source of TMAO production, and studies have shown that higher Firmicutes-to-Bacteroidetes ratios, as observed in red meat and egg-rich diets, appear to be associated with elevated TMAO concentrations. This ecological feedback loop (wherein diet shapes the microbiome, which metabolizes it into TMAO) highlights how dietary interventions can reduce TMAO exposure by limiting both precursor availability and favorable microbial growth conditions, and that dysbiotic communities, which favor TMA production, may directly contribute to inflammation, atherosclerosis (AS), and metabolic impairment independent of TMAO itself.^11^

The choline-to-TMA conversion is mediated by TMA-lyase, an enzyme encoded by the *cutC* gene and regulated by the *cutD* activating gene, via specific bacterial taxa that harbor genes encoding choline TMA-lyase (*cutC*/*D*) and carnitine monooxygenase (*cntA*/*B*) pathways, including the phyla Firmicutes and Actinobacteria, along with species such as *Streptococcus sanguinis, Clostridium* spp. (e.g., *C. difficile, C. asparagiforme, C. hathewayi, C. sporogenes*), *Anaerococcus hydrogenalis*, and genera such as *Escherichia, Klebsiella, Proteus, Providencia, Shigella, Desulfovibrio, Lachnospira, Ruminococcus, Acinetobacter, Serratia, Citrobacter, Edwardsiella tarda, Achromobacter,* and *Sporosarcina.*^12^ Not all of the organisms that produce TMA contribute equally to the TMAO levels in blood. A universal bacterial strain responsible for the elevated TMA/TMAO level does not appear to exist. Instead, TMA production is a functionally redundant process found in several bacterial groups and pathways. The two major known pathways are the choline TMA-lyase complex that is encoded by the *cutC*/*cutD* system and the carnitine oxygenase pathway. Functional analysis of human feces revealed *cutC* in all samples, but its positive organisms were less than 1% of the total community. The *cutC* genes are mostly found in the members of Clostridium XIVa and *Eubacterium sp*. AB3007, while *cntA* is largely found in Gammaproteobacteria, especially *Escherichia coli*, with some representation from *Citrobacter* and *Klebsiella*^13^ The presence of *cutC* does not always correlate with the abundance of a particular bacterium in the community, suggesting that organism abundance alone is not a reliable predictor of bacterial TMA production.^13^ Experimental evidence also supported the claim that TMA is produced at the community level rather than by a specific strain. Colonization with clearly identified human gut bacteria has shown that the presence of such microorganisms may induce TMA production and increase TMAO levels. However, these bacteria make up only a minimal part of the gut microbiota. This suggests that biologically significant factors for TMAO exposure are not the total amounts of particular bacteria but the cumulative quantity of TMA-generating genes produced. This point is crucial when making predictions involving *Clostridium, Providencia*, or *Escherichia*, since the mere presence or increased numbers of a genus do not imply that the taxon can produce TMA.^13^ The relationships between changes in gut flora, the build-up of TMA/TMAO, and illness are puzzling and seem to depend on the type of illness and the conditions at play. Observational studies cannot explain whether more TMA-producing bacteria initiate the disease or result from changes in diet, intestinal physiology, inflammation, medication use, or other metabolic disorders. Experimental studies provide stronger evidence for a causal link between bacterial TMA production, because changing gut bacterial composition or introducing more TMA-producing bacteria changes TMAO levels and illness symptoms in animals. At the same time, the disease could change the gut environment and microbial composition, which could increase activity of TMA-producing pathways. Thus, one could say that there is a mutual relationship between gut flora, TMAO production, and the host's health.^14^, ^15^, ^16^

TMA is then rapidly absorbed across the intestinal epithelium and transported via portal veins to the liver, where it undergoes oxidation, catalyzed by FMO3, which introduces an oxygen atom to form TMAO (Fig. 1), and although liver is the primary site of TMAO synthesis, extrahepatic FMO3 expression has been documented in the kidneys and lungs; however, their quantitative contribution to systemic TMAO remains to be fully characterized.^15^ The FMO family comprises five major isoforms (FMO1–FMO5) capable of oxygenating nucleophilic xenobiotics containing nitrogen or sulfur, as well as certain endogenous substrates in the adult liver.^16^ FMO1 and FMO3 are the primary hepatic isoforms involved in TMA oxidation, with FMO3 demonstrating roughly a tenfold greater specific activity toward TMA than FMO1. The expression of FMO3 is affected by genetic variations and hormonal fluctuations, in which androgens inhibit and estrogens may enhance FMO3 activity, leading to sex-related differences in circulating TMAO levels.^17^

**Fig. 1:**
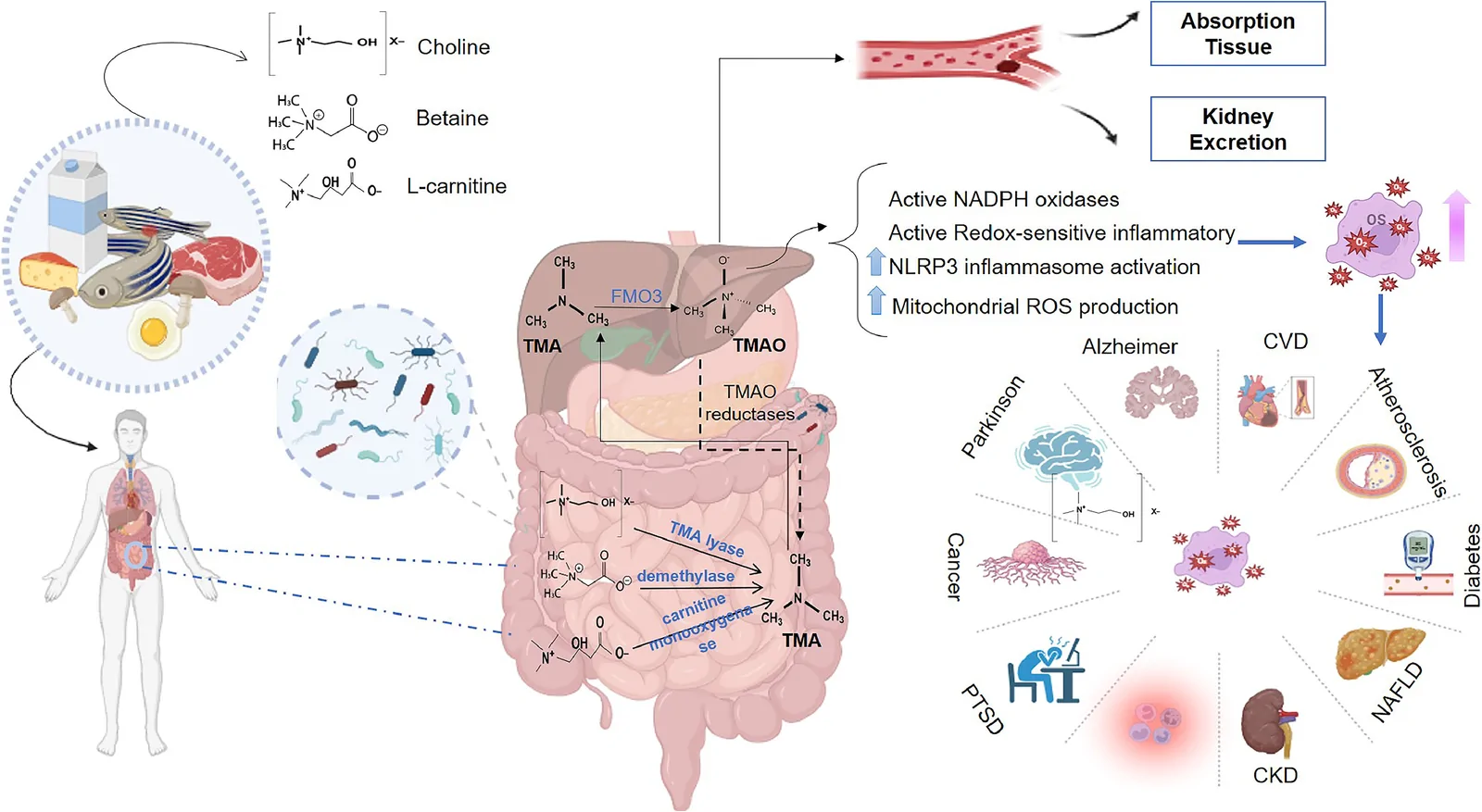
**TMAO metabolic pathway**. *Abbreviations:* CKD, chronic kidney disease; CVD, cardiovascular disease; FMO, flavin-containing monooxygenase; NAFLD, non-alcoholic fatty liver disease; NLRP3, NOD-like receptor protein 3; OS, oxidative stress; PTSD, post-traumatic stress disorder; ROS, reactive oxygen species; TMA, trimethylamine; TMAO, trimethylamine N-oxide.

Renal function is a key variable influencing TMAO clearance rates, as TMAO is predominantly excreted via glomerular filtration, followed by tubular reabsorption and secretion within the nephron, and consequently, diminished renal function, as seen in chronic kidney disease (CKD), leads to decreased TMAO clearance and, predictably, elevated plasma TMAO concentrations.^18^ Furthermore, reports indicate that geriatric populations have a mean TMAO of 6.3 ± 5.8 µmol/L, compared with 1.8 ± 1.4 µmol/L in younger individuals, underscoring compositional shifts in the microbiota toward TMA-producing taxa with aging, alongside age-related declines in renal function.^19^

## 3 Physiological functions of TMAO (Osmoprotection and Protein Stabilization)

TMAO, previously regarded as an inert osmolyte in marine life, is now acknowledged as a metabolite with considerable biological activity that influences mammalian physiology. It is associated with critical processes including metabolism, cardiovascular health, immune function, and neurobiological activities, and this evolving perspective reclassifies TMAO from a mere byproduct to a potential modulator and, in certain contexts, a disruptor of human physiological homeostasis.^20^

TMAO is a natural osmolyte that acts as a chemical chaperone, which stabilizes protein structures following osmotic and chemical stress.^21^ A recent computational study evaluated the role of TMAO as a protein stabilizer and found that this effect peaks at 1 M, beyond which higher TMAO concentrations yield no additional benefit.^21^ Its protective mechanism includes three primary pathways: (1) TMAO has no favorable interactions with the peptide backbone, which will tend to cause it to favor exclusion from the surface as well as from the more folded, compact state; (2) it alters the properties of solvents, such as the structure of water; and (3) TMAO enhances the hydration shell of well-folded proteins, stabilizing and activating the protein in an environment that otherwise promotes denaturation.^22^ At a molecular level, TMAO counteracts the hydrophobic effect of denaturants such as urea and has been suggested to counterbalance urea-induced protein unfolding by increasing the free energy, thereby stabilizing the native state.^23^ In addition, TMAO diminishes water's hydrogen-bonding ability, destabilizing the unfolded state and promoting protein folding, and has been proposed to modulate heat shock protein expression, which is known to contribute to chaperone-assisted protein folding and proteostasis. Furthermore, TMAO is an accumulated compound in organisms in response to environmental pressures in biological systems, as some marine animals raise their tissue levels of TMAO to counteract the destabilizing influence of high hydrostatic pressure and urea, highlighting the importance of TMAO in preserving protein structure and cellular function in the face of stress.^24^

## 4 TMAO-induced pathophysiological mechanisms

### 4.1 Mitochondrial reactive oxygen species (ROS), mitogen-activated protein kinase (MAPK), and inflammasome activation

Mitochondria, nicotinamide adenine dinucleotide phosphate oxidases, and peroxisomes are the primary sources of intracellular ROS, and antioxidants, including superoxide dismutase, catalase, glutathione peroxidase, glutathione, and vitamins C and E, neutralize excess oxidants and maintain redox balance.^25^ TMAO promotes mitochondrial dysfunction and ROS production by upregulating the succinate dehydrogenase subunit B, thereby resulting in increased pore formation on the mitochondrial surface and release of cytochrome c, which then activates caspase-9 and subsequently caspase-3.^26^ Caspase-3 cleaves gasdermin D (GSDMD) at D87 in its N-terminal domain to generate a non-pore-forming fragment, inhibiting pyroptosis and favoring apoptosis, as seen by elevated levels of H₂O₂ and malondialdehyde, indicators of oxidative stress and lipid peroxidation.^27^ This cascade also diminishes ATP synthesis and the ATP/ADP ratio by impairing oxidative phosphorylation.^28^

Concurrently, TMAO-induced ROS production increases nuclear factor kappa-B (NF-κB) and activates protein kinase C in endothelial cells (the initiator of NF-κB activation), increasing inflammasome components including pro-interleukin-1β (pro-IL-1β), pro-interleukin-18 (pro-IL-18), pro-osteogenic genes within the vascular wall, and resulting in osteogenic transformation and vascular calcification, an early stage of atherosclerotic plaque formation, and increasing nucleotide-binding oligomerization domain-like receptor protein 3 (NLRP3) inflammasome, which is a heteromeric proteinaceous complex that plays a central role in the innate immune response to cellular stress and damage-associated molecular patterns (Fig. 2).^29^^,^^30^ Furthermore, TMAO increases thioredoxin-interacting protein dissociation from thioredoxin (TRX) and increases TRX binding to the NLRP3 inflammasome, mediating its assembly with apoptosis-associated speck-like protein containing a CARD (ASC) (ROS- thioredoxin-interacting protein -NLRP3 inflammasome pathway), which directly and indirectly increases mitochondrial reactive oxygen species (mtROS), through inhibition of nicotinamide adenine dinucleotide -dependent deacetylase sirtuin-3 (SIRT3) and superoxide dismutase 2 (SOD2), leading to increased mtROS, a direct activator of the NLRP3 inflammasome (SIRT3-SOD2-mtROS Pathway).^31^ Activated NLRP3 inflammasome then activates caspase-1, thereby promoting pro-inflammatory cytokine maturation, including pro-IL-1β and pro-IL-18, and cleaves GSDMD at D275, resulting in increased pyroptosis, increased cytokine release, and vascular inflammation, endothelial permeability, and dysfunction, all of which are known pro-atherogenic factors.^32^ Parallel to the NF-κB/NLRP3 pathway, TMAO recruits leukocytes to endothelial cells and activates the MAPK) pathway, a pivotal pro-inflammatory pathway due to its role in increased transcription of tumor necrosis factor (TNF)-α and NLRP3 inflammasome, and is responsible for survival, cell proliferation, and differentiation, and regulation of apoptosis, which consists of four major pathways, including the extracellular signal-regulated kinases (ERK) 1/2, ERK5, c-Jun N-terminal kinase, and p38 MAPK signaling pathway.^33^ TMAO-induced ROS activates MAP3Ks, which then phosphorylate MAP2Ks, which subsequently phosphorylate MAPKs.^33^^,^^34^ Simultaneously, ROS induces activation of both ERK1/2 and Jun N-terminal kinase MAPK signaling pathways; the latter, like p38, is associated with increased transcription of pro-inflammatory cytokine genes, leading to endothelial dysfunction, atherogenesis, thrombosis, and AS.^33^

**Fig. 2:**
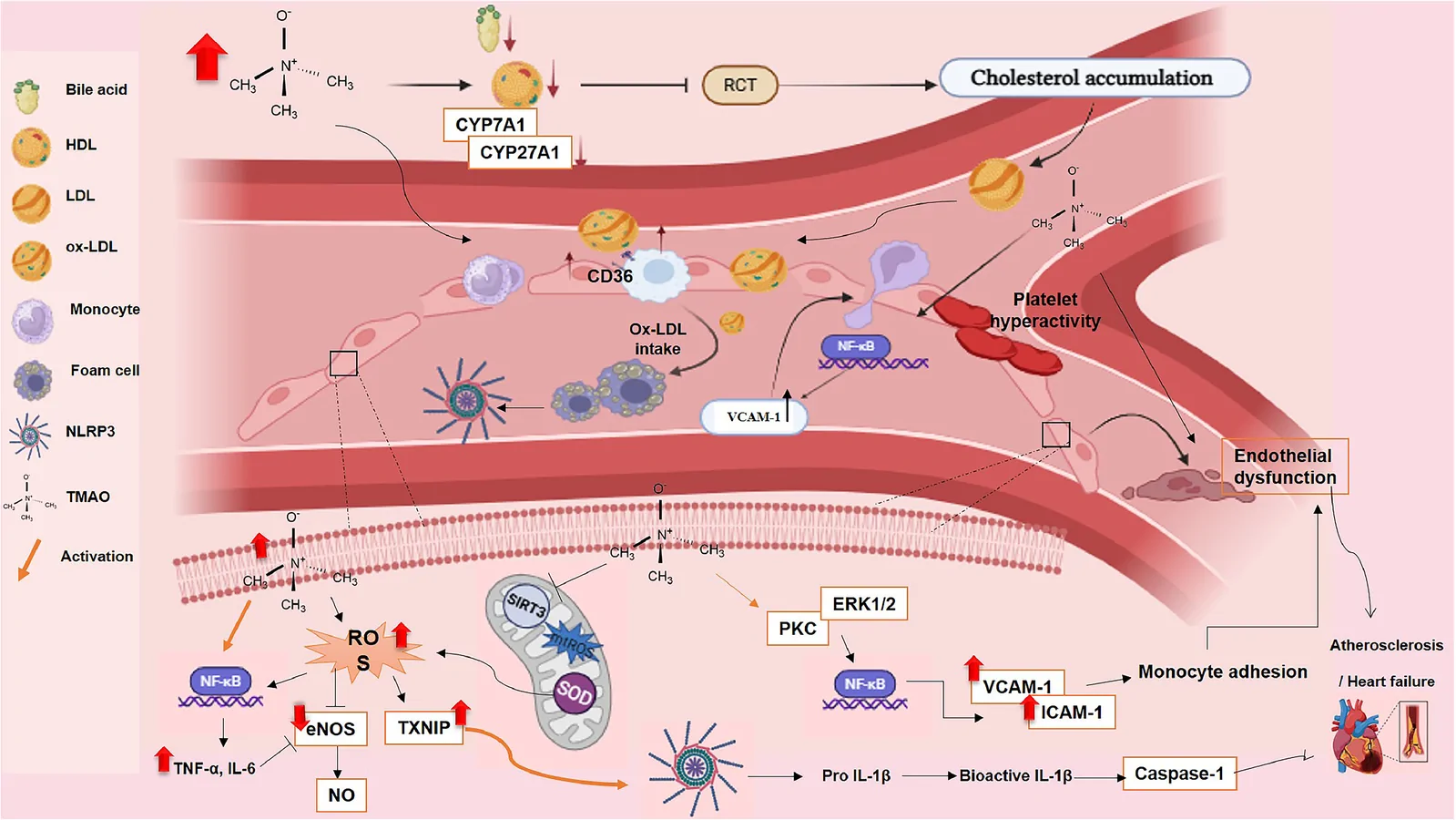
**Pathogenic mechanisms of TMAO.***Abbreviations:* CD, cluster of differentiation; eNOS, endothelial nitric oxide synthase; ERK, extracellular signal-regulated kinases; GSDMD, gasdermin D; GVHD, graft versus host disease; HDL, high-density lipoproteins; IL, interleukin; LDL, low-density lipoproteins; NF-κB, nuclear factor kappa-B; NLRP3, NOD-like receptor protein 3; NO, nitric oxide; ox-LDL, oxidized LDL; SIRT3, sirtuin-3; SOD2, superoxide dismutase 2; TMA, trimethylamine; TMAO, trimethylamine N-oxide; TNF, tumor necrosis factor; TXNIP, thioredoxin-interacting protein.; VCAM-1, vascular cell adhesion molecule; ICAM-1, intercellular adhesion molecule; PKC, protein kinase C.

Additionally, TMAO independently activates Toll-like receptor (TLR) 4, a pattern recognition receptor expressed on the surface of macrophages, dendritic cells, enterocytes, and endothelial cells, the intracellular domain of TLR4, upon its activation, induces recruitment of the molecule myeloid differentiation primary response gene 88 to the intracellular domain, which then recruits the interleukin (IL)-1 receptor-associated kinase (IRAK) 1, IRAK-2, and IRAK-4, eventually resulting in the phosphorylation of the inhibitor of kappa B kinase-β, which normally inhibits NF-κB nuclear translocation.^35^ Phosphorylation of inhibitor of kappa B kinase-βremoves this inhibitory effect, resulting in NF-κB nuclear translocation and promoting pro-inflammatory cytokine production.^36^

While some studies report minimal impact on mitochondrial membrane potential in certain cell types, others demonstrate marked depolarization alongside apoptotic activation, suggesting TMAO's pro-oxidant effects are cell-type dependent.^37^ In most cells, the caspase-3/GSDMD-D87 pathway dominates, resulting in apoptosis, whereas in some, the NLRP3/caspase-1/GSDMD-D275 pathway is prioritized, resulting in increased pyroptosis.^38^ A smaller number of cells undergo mixed cell deaths, including both apoptosis (endothelial damage) and pyroptosis (vascular damage via IL-1β).^38^ Combined, TMAO enhances ROS generation, triggering inflammatory signaling cascades and disrupting mitochondrial function, all of which lead to cellular and tissue impairment in pathogenic conditions.

### 4.2 Endothelial dysfunction

Endothelial dysfunction, a hallmark of cardiovascular disorders and AS, is the result of a mismatch between vasoconstrictive and vasodilatory mechanisms and is characterized by reduced nitric oxide (NO) bioavailability, which is involved in vascular tone regulation.^39^ Whether TMAO influences NO production at physiological levels is debated, as a recent translational study reported neutral effects of TMAO at physiological levels on endothelial nitric oxide synthase (eNOS), the primary source of NO, in contrast to high levels, which may directly alter NO production through competitive inhibition of eNOS, highlighting its dose-dependent suppressive effects on NO production, which results in endothelial inability to initiate vasodilation.^40^^,^^41^

Physiologically, eNOS uses tetrahydrobiopterin as a cofactor to convert L-arginine to NO. In the presence of increased TMAO-induced ROS, tetrahydrobiopterin is oxidized to dihydrobiopterin and a transient trihydrobiopterin radical, resulting in eNOS uncoupling and production of superoxide (O₂⁻) instead of NO, in addition to increased L-arginine efflux, the substrate for eNOS.^42^ The increased superoxide then reacts with residual NO and forms peroxynitrite (ONOO⁻), which oxidizes low-density lipoproteins (LDLs) and reduces glutathione, and nitrates SOD, inhibiting cellular defense against increased ROS, and forming oxidized LDL (ox-LDL), further resulting in a vicious cycle of excess ROS production.^43^

Activation of the NF-κB and MAPK pathways also increases endothelin-1 mRNA expression and production, a major regulator of vasoconstriction, via activation of inositol trisphosphate-dependent Ca^2+^ release, further promoting contractility and endothelial dysfunction.^39^ One study demonstrated that TMAO elevation *in vivo* and *in vitro* induced increased cathepsin B, which directly promotes NLRP3 inflammasome activation and results in increased IL-1β, disrupting inter-endothelial junctions and leading to increased endothelial permeability, and another *in vitro* study reported that treatment of human umbilical vein endothelial cells with 100 µmol/l of TMAO, in a dose-dependent manner, resulted in a 1.20-fold increased expression of vascular adhesion molecule 1 via NF-κB activation from Section 4.1 pathways, a well-established indicator of endothelial injury.^44^^,^^45^

### 4.3 Endoplasmic reticulum stress

The endoplasmic reticulum (ER) is an organelle responsible for lipid metabolism, Ca²⁺ homeostasis, and protein folding and synthesis. Disruption in its function can result in the accumulation of misfolded and unfolded proteins, the unfolded protein response, and eventually, ER stress.^46^ Unfolded protein response, crucial to proteostasis restoration in acute stress, consists of three signaling pathways: Protein kinase R-like ER kinase (PERK), activating transcription factor (ATF) 6, and inositol-requiring transmembrane kinase/endoribonuclease-1α (IRE1α). In acute stress, all branches are activated, and eIF2α is phosphorylated via PERK, which then prevents protein overload in the ER and increases translation of ATF4.^47^ ATF4’s interaction with the CCAAT/enhancer-binding protein homologous protein then results in the promotion of target genes’ expression, indirectly recovering protein synthesis.^46^ Conversely, in sustained ER stress, as observed in increased TMAO levels, CCAAT/enhancer-binding protein homologous protein upregulates pro-apoptotic genes, and increased TMAO levels preferentially activate the PERK pathway, and PERK directly induces eIF2α hyperphosphorylation.^46^^,^^47^ This PERK dominance under chronic TMAO stress explains the shift toward apoptosis rather than adaptive IRE1/ATF6 responses.

### 4.4 Lipid dysregulation

TMAO disrupts lipid metabolism primarily by promoting foam cell formation, impairing cholesterol efflux, and enhancing lipid accumulation in macrophages and vascular cells.^48^ The primary pathway leading to increased foam cell formation is upregulation of CD36, which increases ox-LDL uptake by macrophages.^48^ Moreover, TMAO upregulates heat shock protein 70 (glucose-regulated protein 78), further increasing lipid uptake.^48^ Reverse cholesterol transport (RCT) is the process of transferring excess cellular cholesterol to the liver for its excretion as bile acid or neutral sterols.^48^ One *in vivo* study specifically evaluated the role of TMAO in RCT inhibition and reported that choline- or L-carnitine-fed mice demonstrated approximately 30% lower RCT than controls. In contrast, TMAO-fed mice exhibited a 35% decrease in RCT.^49^ However, they observed that TMAO modestly decreased cholesterol transporters compared with bile acid transporters, whose mRNAs decreased significantly in TMAO-fed mice, suggesting that the bile acid synthetic pathway may play a major role at multiple levels.^49^ Studies indicate that TMAO disrupts cholesterol homeostasis and bile acid metabolism primarily by suppressing hepatic bile acid synthesis, rather than by directly reducing bile acid pool volume. This suppression reduces cholesterol conversion into bile acids, thereby limiting fecal sterol excretion and contributing to increased plasma LDL cholesterol.^50^^,^^51^ The underlying molecular mechanism involves TMAO-mediated activation of the small heterodimer partner and farnesoid X receptor nuclear receptors. This signaling cascade subsequently downregulates cholesterol 7α-hydroxylase, the rate-limiting enzyme in the classical bile acid synthetic pathway (Fig. 2).^50^^,^^51^ This suppressed synthesis decreases overall bile acid production, reducing cholesterol clearance and promoting a pro-atherogenic lipid profile.

### 4.5 Platelet hyperactivity

Platelet aggregation and hyperactivity are associated with increased risk of thrombotic events and end-organ injury, which can occur in hyperlipidemia, increased ROS, and hyperglycemia.^52^ *In vivo* studies have demonstrated increased risk of thrombotic events with increasing plasma TMAO levels, in a dose-dependent manner, and through rapid platelet activation, which is crucial for thrombosis development.^52^ G-protein-coupled receptors influence this process; they are the largest receptor family in humans. TMAO can increase G-protein-coupled receptors signaling, thereby promoting inositol trisphosphate release and receptor activation on the dense tubular system, resulting in cytosolic Ca^2+^ mobilization. Cytosolic Ca^2+^ then induces increased thromboxane A2 and ADP release, activates myosin light chain kinase, phosphorylates myosin light chains, and results in actin-myosin contraction, exposing glycoprotein receptors for fibrinogen binding, and enabling irreversible platelet aggregation.^52^

## 5 TMAO in disease pathophysiology

The context-dependent role of TMAO in various diseases has been extensively studied, and multiple disorders have been linked to this gut microbiota metabolite. The main pathogenesis pathways behind how TMAO contributes to such disorders are related to the role of TMAO-induced ROS and pro-inflammatory cytokine production, resulting in increased inflammation, both chronic and acute, and thus, involvement of almost every organ, including the kidneys, pancreas, central nervous system (CNS), cardiovascular system, and gastrointestinal system.^53^ Additionally, TMAO influences the immune system and can modulate both innate and adaptive immune responses.^53^

### 5.1 AS

Although multifactorial, inflammation and immune system activation are believed to play major roles in AS pathogenesis through lipid accumulation, increased oxidative stress, recruitment of immune cells, and increased foam cell formation by ox-LDL ingestion, thereby forming atherosclerotic plaque and resulting in endothelial dysfunction, all of which can occur as a result of increased TMAO circulation, as discussed earlier.^54^ One recent multi-ethnic study aimed to evaluate the association between TMAO and ASCVD and observed that higher TMAO plasma levels were significantly associated with a 9% (hazard ratio [HR]: 1.09, 95% confidence interval [CI]: 1.03–1.15) and 32% (HR: 1.32, 95% CI: 1.01–1.73) higher risk of ASCVD in continuous (median of the fifth *vs.* first quintiles) and quintile analyses (Q5 *vs.* Q1), respectively.^55^ Furthermore, another study supported their findings by demonstrating that TMAO was associated with a 2.86-fold (95% CI: 1.82–3.97, Q4 *vs.* Q1) increased mortality risk in patients with AS and peripheral artery disease, corroborated by Li et al.’s findings,^45^^,^^56^ who reported an increased significant risk of 7-year mortality rate in individuals with higher TMAO levels (Q4 adjusted HR: 1.81, 95% CI: 1.04–3.15). In addition, TMAO plasma levels positively correlate with atherosclerotic plaque size in animal models, and dietary TMAO supplementation in mice (*ApoE*^−/−^) induced aortic atherosclerotic lesions in one study.^15^ Given the embodied evidence of the association between elevated TMAO and increased atherosclerotic plaque formation, emerging gut microbiota-targeted strategies should be aimed at TMAO plasma level modulation, especially in individuals with an underlying CVD, including dietary implementation of probiotics, prebiotics, synbiotics, and postbiotics to restore healthy gut flora.

### 5.2 Hypertension (HTN)

Recently, a direct and linear dose-dependent association between plasma concentrations of TMAO and HTN, heavily influenced by lifestyle habits, was revealed, as a meta-analysis reported that individuals with CVD were 14% at higher risk of developing HTN, and this risk increased by 1.014% per 1 µmol/L increase in TMAO concentrations,^57^ which was further corroborated by a clinical study that demonstrated that a 1-unit increase in TMAO resulted in a 1-standard deviation increase in systolic blood pressure (β: 0.039, SE: 0.072).^58^ Such an association can be attributed to TMAO's role in prolonging the hypertensive effects of angiotensin II, increasing vasoconstriction, water reabsorption, and arterial stiffness via increased ROS, thereby increasing blood flow velocity and HTN.^59^ One study demonstrated that TMAO positively correlated with increased systolic blood pressure in healthy middle-aged to older individuals and elderly mice via advanced glycation end-products and superoxide-stimulated oxidative stress production, and comparably, a systematic review and meta-analysis observed that high plasma TMAO levels were significantly correlated with increased risk of HTN (relative risk: 1.12, 95% CI: 1.06–1.17), as the risk increased by 9% per 5-µmol/L increment (relative risk: 1.09, 95% CI: 1.05–1.14) and 20% per 10-µmol/L increment of TMAO levels (95% CI: 1.11–1.30).^19^^,^^60^ However, given the impact of gut dysbiosis on both TMAO and vascular function, and despite multiple studies highlighting the effects of TMAO on BP, failure to account for microbiome alterations limits the interpretation of direct causal effects of TMAO on HTN.^61^ A recent study also reported that the association between TMAO and HTN may be age-dependent, and that younger individuals with higher TMAO concentrations may benefit more from TMAO-lowering pharmaceuticals, thereby reducing their HTN risk in old age.^62^

### 5.3 Heart failure (HF)

Greater blood levels of circulating TMAO have been associated with an increased risk of AS, HF, and thrombotic events, potentially due to endothelial dysfunction, platelet hyperactivity, and vascular inflammation.^63^ A large-scale multi-ethnic study on 11,768 patients over a median follow-up of 15.9 years reported higher levels of TMAO were significantly associated with increased incidence of HF (HR: 1.15, 95% CI: 1.09–1.20), possibly via reduced blood supply to the gut, which then impairs the gut barrier, increases gut permeability, alters gut microbiota composition, and induces chronic systemic inflammation.^63^ In addition, TMAO may contribute to ventricular remodeling and myocardial fibrosis via the *Smad3* signaling pathway, increased mtROS, and endothelial cell inflammation, as TMAO-depleted diets in mouse models improved cardiac function and mitigated adverse ventricular remodeling.^64^^,^^65^ Furthermore, heart failure patients exhibiting elevated plasma TMAO concentrations may face an increased risk of developing left ventricular diastolic dysfunction and long-term mortality, as significant correlations have been identified between high TMAO levels, cardiovascular events, and a poorer one-year prognosis in individuals with cardiovascular disease, as well as a rise in post-discharge cardiovascular events among HF patients with elevated TMAO levels.^66^^,^^67^

### 5.4 Neurodegenerative diseases

#### 5.4.1 Alzheimer's disease

Recently, the possible role of TMAO in AD progression and incidence is emerging, as one animal study demonstrated that TMAO exposure resulted in impaired working memory and increased escape latency in the Morris water maze, implicating learning and spatial memory impairments, in addition to Vogt et al., who observed higher TMAO concentrations in the cerebrospinal fluid of individuals with mild cognitive impairment ([2.1 ± 1.4] scaled intensity units) compared to controls ([1.3 ± 1.5] scaled intensity units), indicating blood-brain barrier (BBB) permeability to TMAO.^68^^,^^69^ Physiological levels of TMAO within cerebrospinal fluid have been reported to increase BBB integrity against inflammation through restriction of astrocyte and microglial reactivation, in contrast to pathologically high TMAO levels, which can induce CNS inflammation, disrupt BBB integrity via downregulation of tight junction proteins, and further increase cognitive impairment.^70^ One review also suggested that the accumulation of Aβ peptide and tau protein is associated with increased circulating TMAO.^71^ Physiologically, the tau protein helps stabilize neuronal microtubules, and the Aβ peptide plays a major role in neuronal plasticity, which results in improved synaptic development.^72^ In AD, however, soluble Aβ hyperphosphorylates tau protein, increases ROS production and inflammatory responses, aggregates into neurofibrillary tangles within neurons, and ultimately leads to neuronal death, a process that may be exacerbated by elevated circulating TMAO levels.^73^ As this metabolite sensitizes neuroinflammatory signaling, particularly in the presence of systemic metabolic dysregulation, important questions about its long-term CNS effects arise.

#### 5.4.2 Parkinson's

Although evidence suggests that gut microbiota alterations increase α-synuclein misfolding, a key pathological driver of Parkinson’s disease (PD), ongoing debates persist about whether TMAO increases PD incidence and alleviates its signs and symptoms, as various studies have reported contradictory results. One study observed higher TMAO concentrations in patients with advanced PD, particularly in individuals with motor fluctuations.^74^ Furthermore, circulating TMAO levels were found to be significantly lower in individuals at the early stages of PD, and patients with TMAO levels below 4.75 µmol/L were more resistant to lower doses of dopaminergic medication; additionally, other studies have reported that TMAO stabilizes α-synuclein peptide conformations, potentially inhibiting its pathogenic aggregation and misfolding.^75^^,^^76^ In addition, Xu et al. observed higher plasma TMAO concentrations in those with PD and cognitive impairment (median: 137.5 ng/mL, IQR: 105–175 ng/mL), compared to PD patients without cognitive impairment (median: 131.5 ng/mL, IQR: 93.8–159 ng/mL) and healthy controls (median: 101 ng/mL, IQR: 79.3–114 ng/mL).^77^ Furthermore, orally induced high levels of TMAO in mouse models resulted in increased microglial and astrocytic activation in the striatum and substantia nigra, aggravating PD-related brain pathology.^78^ Although still unclear, it is hypothesized that TMAO exposure disrupts the BBB, increasing permeability and microglial activation and inducing neuroinflammation via the NLRP3 inflammasome, in addition to aggravating β-amyloid or α-synuclein pathology, as a result of increased TMAO concentrations.^79^

### 5.5 Metabolic disorders

#### 5.5.1 Diabetes

Type 1 diabetes involves autoimmune β-cell destruction, while type 2 Diabetes mellitus (T2DM) commonly features peripheral insulin resistance (IR) and progressive β-cell loss, driven by oxidative stress and chronic inflammation.^28^ One significant cytokine in IR is TNF-α, which exerts its effects by decreasing glucose transport inside cells.^80^ Although the role of oxidative stress in IR is well established, conflicting evidence exists regarding the association between TMAO and IR, as one study reported increased TMAO levels after initiation of metformin treatment. In contrast, others reported that metformin suppressed circulating TMAO, impaired glucose tolerance, and reduced glucose-stimulated insulin secretion.^28^^,^^81^ Notably, one recent study found that TMAO was significantly associated with T2DM risk only at its highest quartile (odds ratio: 3.35, 95% CI: 1.55–7.26), with a decreasing and then increasing (U-shaped) trend in TMAO levels in newly diagnosed patients with T2DM.^82^ Additionally, Li et al. demonstrated that oral administration of TMAO, not intraperitoneal injection, impaired glucose tolerance.^83^ This finding was likely confounded by the conversion of TMAO to TMA upon oral administration, which does not occur with intraperitoneal injection.^84^ Moreover, the beneficial roles of TMAO in diabetic mice in preventing diabetic nephropathy and normalizing glucolipotoxic-mediated damage in pancreatic β-cells and primary islet function have been noted, contrasting with another *in vivo* study, which reported an increased incidence of IR in monkeys fed with a TMAO-rich diet compared with a regular diet, possibly due to impaired hepatic glucose transportation and increased gluconeogenesis, necessitating further research to conclude the exact role of TMAO in diabetic patients.^85^^,^^86^

#### 5.5.2 Non-alcoholic fatty liver disease

Non-alcoholic fatty liver disease (NAFLD), a common and prevalent cause of chronic liver disease, is characterized by hepatic steatosis in the absence of any other contributing factor, to which various factors, including inflammatory processes and ROS accumulation, have been attributed.^87^ Recent studies have evaluated the interaction between NAFLD incidence and TMAO and reported an approximate two-fold increase in NAFLD incidence risk for every unit increase in circulating TMAO levels, along with increased risks of all-cause mortality and liver steatosis progression.^87^^,^^88^ Furthermore, in a recent systematic review and meta-analysis, patients with NAFLD had higher plasma TMAO concentrations compared to controls (standard mean difference: 0.43, 95% CI: 0.02–0.85), consistent with another study, where a steatosis score ≥ 1 was associated with an odds ratio of 3.58 per 1 unit increase in ln-transformed TMAO (95% CI: 1.38–9.26), and higher urinary TMAO concentrations have been reported to be associated with a higher risk of IR and NAFLD in animal models.^89^^,^^90^ Moreover, as previously discussed, TMAO can increase inflammation, disturb hepatocyte lipid metabolism, reduce the bile acid pool, and increase oxidative stress, all of which are attributed to NAFLD incidence.^87^

### 5.6 Cancers

Although evidence is emerging on the potential association between TMAO and cancers, a genome-based evaluation indicated that TMAO may contribute to the incidence of colorectal cancer (CRC), further supported by other studies’ findings, demonstrating significant associations between TMAO and its precursor, TMA, and CRC, and a worse overall prognosis in cancer patients with higher TMAO concentrations.^91^ By comparison, Bae et al.^92^ reported a 3.38-fold (95% CI: 1.25–9.16) increased risk of CRC in American women in the highest TMAO quartile compared to the lowest quartile, consistent with the 1.90-fold (95% CI: 1.24–2.92) increased risk of distal CRC observed by Byrd et al.^93^ Additionally, despite the evidence that chronic inflammation within the vascular endothelium, as observed in increased TMAO concentrations, promotes angiogenesis, tumor initiation and progression, and increases vascular dysfunction and permeability, which may increase metastasis risk, two independent studies reported on the anti-tumor properties of TMAO and proposed that TMAO may enhance T cell function, activate myeloid cells, induce pyroptosis in tumor cells, regulate tumor growth, and increase response to treatment in individuals with pancreatic ductal adenocarcinoma and triple-negative breast cancer.^94^^,^^95^

### 5.7 Chronic kidney disease

In 2006, Bain et al.^96^ evaluated the association between TMAO and CKD and reported a possible connection, as patients with CKD on hemodialysis had higher circulating levels of TMA and TMAO; Tang et al.^97^ extended this, concluding that TMAO could increase long-term mortality and CKD progression. In 2016, researchers discovered an association between the FMO3 genotype at amino acid 158, all-cause mortality, disease progression, and circulating TMAO in plasma.^98^ Sun et al.^17^ reported the role of TMAO-induced inflammation in CKD pathogenesis, observing kidney damage due to increased TMAO in high-fat diet-fed mice. Lee et al.^99^ observed that AKI-to-CKD progression was alleviated in the presence of TMAO, with NOX2 identified as the key TMAO regulator. Furthermore, the correlation between TMAO and higher mortality risk in CKD patients appears to be dose-dependent.^100^

Kidneys are documented as the main route of TMAO excretion, indicating that TMAO levels may increase in the presence of CKD, possibly further contributing to CKD progression.^101^ Although the definite mechanism by which TMAO exerts its effects on CKD remains to be fully understood, several studies have proposed that TMAO may promote CKD by stimulating pro-inflammatory cytokine production, including IL-6 and TNF-α, inhibiting nitric oxide production, disrupting sodium and water reabsorption, and increasing oxidative stress and smooth muscle cell proliferation.^102^^,^^103^ Building on the previous evidence, the association between CKD and TMAO likely represents a bidirectional gut–kidney axis, in which kidneys become increasingly incapable of excreting TMAO, leading to TMAO accumulation; elevated TMAO levels, in turn, exacerbate renal inflammation, fibrosis, and functional decline.

### 5.8 Neuropsychiatric disorders

The possible correlation between TMAO and neuropsychiatric disorders within the literature is limited, as only a few studies have been conducted aiming to evaluate this association. Baranayi et al.^34^ evaluated the biomarker role of TMAO in post-traumatic stress disorder and post-acute myocardial infarction. They reported higher TMAO concentrations in those with acute myocardial infarction -induced post-traumatic stress disorder symptoms.^34^ Moreover, TMAO may exacerbate depressive-like behavior in the post-stroke depressive mice model through activation of the ROS-p38/MAPK pathway and increase TNF-α and IL-1β in the hippocampal CA3 region.^104^^,^^105^ One study^106^ also demonstrated that individuals with anxiety disorder exhibited lower plasma TMAO concentration, and another reported TMAO pathway alterations in individuals with schizophrenia, possibly indicating clinical importance in this disorder.^107^

### 5.9 Inflammatory and autoimmune diseases

Researchers have examined whether TMAO exacerbates inflammatory and autoimmune diseases across various disorders. One such is Crohn’s disease, a chronic inflammatory bowel disease (IBD) characterized by abdominal pain, diarrhea, fatigue, and weight loss.^108^ One study reported that individuals with IBD (2.27 µM lower) and active ulcerative colitis disease (1.84 µM lower) had lower plasma TMAO concentrations than controls, with no significant difference between active and inactive Crohn’s disease patients.^109^ TMAO may be inversely associated with IBD risk, and dysbiosis may occur in Crohn’s disease patients with significantly lower TMAO levels than healthy controls.^108^ Given the inflammatory nature of rheumatic disorders, including juvenile idiopathic inflammatory myopathies and systemic lupus erythematosus, researchers have speculated that gut dysbiosis may trigger disease activity and exacerbate disease severity.^110^ Studies have reported elevated urinary TMAO concentrations in patients with juvenile idiopathic inflammatory myopathy and suggested that these concentrations may correlate with the severity of psoriatic arthritis, in both skin and joint manifestations.^111^^,^^112^ Additionally, TMAO may induce autoimmunity by activating T helper 17 cells and increasing TLR7 expression; after TMA inhibition, anti-dsDNA antibody levels in patients with systemic lupus erythematosus decrease.^113^^,^^114^ Contradictory results can be observed on the association between TMAO and graft versus host disease (GVHD), as murine models suggest that increased vascular inflammation due to increased circulating TMAO may have a role in triggering GVHD, possibly following increased M1-like cytokine secretion from macrophages of bone marrow, contradicting another study that reported increased NLRP3 inflammasome following increased TMAO levels may reduce GVHD severity, warranting more research on the interaction between TMAO and GVHD.^115^^,^^116^

## 6 Determinants of the protective and detrimental effects of TMAO

Whether TMAO truly contributes to disease progression or not remains incompletely understood. Although existing evidence largely points to a detrimental role for TMAO, growing evidence also supports its beneficial effects. For instance, one study reported an inverse correlation between TMAO levels and aortic lesion sizes in the thoracic aorta and aortic roots in *ApoE*^−/−^mice. Furthermore, the study documented for the first time that TMAO may not affect foam cell formation, as there were no changes in acetylated LDL cholesterol loading or cholesterol efflux following treatment with TMAO.^117^ Additionally, another study documented no changes in blood pressure parameters following a 2-week 100-fold increase in circulating TMAO levels in normotensive rats.^118^ These findings suggest that TMAO’s pathogenicity is not universal and that it may depend on host immune and metabolic status, tissue type, disease stage, and exposure characteristics.

Emerging evidence supports this view, as TMAO has been demonstrated to induce antitumor effects through increasing triple-negative breast cancer cells’ pyroptosis via the PERK pathway, contrasting the protumor effects observed in CRC cells, emphasizing that its effects are context-dependent.^119^ Furthermore, the circulating levels of TMAO vary greatly between individuals, with a reported median of 3.45 µM, and indeed the concentrations used in *in vitro* models to induce pathological responses are not often observed in *in vivo* models.^120^^,^^121^ Collectively, the net effect of TMAO likely depends on concentration, exposure duration, tissue susceptibility, gut microbiota composition, and the host's baseline inflammatory and metabolic status, as transient exposures or low concentrations may support adaptive proteostasis and stress responses. In contrast, chronic exposure and high doses could promote oxidative stress and further contribute to inflammation and endothelial dysfunction.

## 7 Beneficial roles of TMAO

Imbalances within the ER, a vital organelle for protein folding, including protein mutations, can saturate ER protein-folding capacity and thereby induce ER stress, which plays a critical role in the pathogenesis of various diseases.^46^ Most recently, TMAO has attracted growing attention for its protein-stabilizing capabilities, as observed in ER stress, and for enhanced protein stability against thermal stress and denaturation.^122^ Moreover, one study reported reduced mitochondrial energy metabolism, resulting in restoration of ventricular function and decreased brain natriuretic peptide in TMAO-treated compared with untreated rats, suggesting that increased TMAO concentrations impeded the metabolic shift observed in ventricular dysfunction by inhibiting β-oxidation under stress conditions.^123^ Additionally, one recent study reported that increased TMAO concentrations by 3–5-fold induced natriuresis and diuresis, lowered diastolic BP in rats with HF, and reduced mortality, implicating that TMAO's adverse effects are linked to its precursor, TMA.^124^ Moreover, other studies suggest that TMAO may also reduce cardiac fibrosis and improve hemodynamics in hypertensive rats, in addition to slowing aortic lesion formation, as chronic low-dose TMAO treatment exacerbated hypertension-related symptoms. In contrast, higher concentrations were not associated with the development of hypertension.^125^^,^^126^ TMAO may act as an osmoprotectant against hyperammonemia and glutamate toxicity in neurons, and it may promote tau-tubulin assembly by lowering the critical tubulin concentration and potentially preventing tau aggregation.^127^ TMAO levels have also been reported to reduce cholesterol absorption, and its effects under stress conditions appear to differ from those in healthy individuals, as TMAO has been shown to improve insulin sensitivity under ER stress.^50^ A similar finding was observed by Dumas et al.^128^^,^^129^ as they reported enhanced glucose tolerance in mice fed a high-fat diet, and that TMAO may induce insulin secretion in isolated pancreatic islets, corroborated by the findings of another study, where TMAO maintained pancreatic β-cell health as well as decreased ER stress markers, suggesting cytoprotection in glucolipotoxic conditions.^128^^,^^129^ Additionally, one study reported that TMAO has therapeutic efficacy regarding epidermolysis bullosa by protecting keratinocytes from keratin aggregation.^130^ Additionally, TMAO may enhance wound healing by inhibiting neutrophil extracellular traps and promoting angiogenesis.^131^ However, TMAO's antioxidative function is dose- and context-dependent. In particular, in pathological conditions such as vascular endothelial dysfunction, TMAO has been found to impair mitochondrial antioxidant defenses. Chen et al. found that TMAO repressed SIRT3 expression, reducing SOD2 activity and thereby potentiating mitochondrial ROS accumulation. These observations complicate TMAO's function, indicating that moderate concentrations support antioxidant defense mechanisms, whereas excessive or chronic exposure shifts the balance toward oxidative stress.^132^ In summary, TMAO plays a dual role in redox biology. Depending on cellular context, concentration, and environmental stressors, TMAO may act as an antioxidant enhancer or a pro-oxidant. The reverse functionality should be emphasized to highlight the roles of physiological thresholds and cellular sensitivity in understanding TMAO's effects on oxidative stress.

## 8 Therapeutic strategies targeting TMAO metabolism

### 8.1 Dietary and microbiota-directed interventions

#### 8.1.1 Prebiotics and probiotics

Prebiotics are non-digestible substrates that can promote proliferation of beneficial bacteria following fermentation by gut microbiota, which a systematic review and meta-analysis reported may reduce serum TMAO levels.^133^ However, substantial heterogeneity limits the generalizability of these findings. Recently, a study by Chen et al. demonstrated that resveratrol, a microbiota-modulating polyphenol which is commonly found in grapes and berries, attenuated TMAO-induced AS in ApoE^−;/−^ mice via inhibition of TMAO production, an effect which was abolished following antibiotic exposure.^51^ Furthermore, probiotics are live strains of microorganisms that can confer a health benefit on the host when administered in adequate amounts. To evaluate the effects of probiotics on TMAO levels, a study conducted in 2015 reported that 4 weeks of treatment with Bifidobacterium (630 mg per intake twice a day) significantly reduced TMAO levels in patients with unstable angina, corroborated by Spasova et al.'s study,^134^^,^^135^ which documented that 12 weeks of Lactobacillus plantarum supplementation significantly reduced serum TMAO levels from 284 µg/L to 202.5 µg/L in patients with a history of atherosclerotic cardiovascular disease. However, a recent systematic review and meta-analysis of randomized controlled trials reported that probiotics have no significant effects on TMAO levels compared to controls (weighted mean difference = − 0.08 µmol/L; 95% CI : −0.76–0.60; *p* = 0.813).^136^ Collectively, the exact effect of probiotics on TMAO levels appears inconsistent and requires further large-scale randomized controlled trials to elucidate their effects better.

#### 8.1.2 Gut microbiota remodeling

In addition to probiotics and prebiotics, dietary modifications and microbiota transplantation may also alter TMAO levels, as demonstrated by a randomized controlled trial in which short-term adherence to a Mediterranean diet significantly reduced circulating TMAO levels in individuals with normal or overweight body mass index.^137^ Additionally, parallel findings were observed by another study, reporting reductions in TMAO levels at weeks 1 and 8 after 8 weeks of a vegan diet, which was followed by 4 weeks of unrestricted diet that resulted in increases in TMAO levels at week 12.^8^ Moreover, fecal microbiota transplantation targets the host microbiome, aiming to restore eubiosis; the effects of which on TMAO levels are not accurately determined, as only one double-blind randomized controlled trial aimed to evaluate whether fecal microbiota transplantation from vegan donors affected TMAO levels, which reported that despite detectable changes in microbiota composition, TMAO production capacity remained unchanged.^138^

### 8.2 Targeted inhibition of microbial TMA production

TMA lyase is an enzyme that catalyzes the conversion of dietary choline to TMA, the immediate precursor of TMAO; targeting this pathway may reduce circulating TMAO levels.^139^ Iodomethylcholine (IMC) is a non-lethal, time-dependent, irreversible inhibitor of bacterial *CutC*/*D* enzymes that eventually blocks dietary choline conversion to TMA and, subsequently, TMAO; limited exposure in mice has been reported to effectively reduce TMAO levels for a prolonged period.^140^ Furthermore, one study evaluated the effects of IMC on choline diet-induced reductions in glomerular filtration rate (GFR) and increases in urinary albumin-to-creatinine ratio, which were completely reversed following IMC administration in the isoproterenol infusion model, corroborated by another study, which evaluated the effects of a 6-week IMC-containing diet in male mice, initiating 3 weeks before transverse aortic constriction. Their findings documented improved cardiac remodeling and function compared with choline-fed mice.^65^^,^^141^ Although a growing body of evidence points to a potential benefit of inhibiting TMA lyase activity, a significant gap remains in the administration of IMC and similar compounds in humans. Thus, future studies should evaluate the efficacy and safety of orally bioavailable inhibitors in clinical trials across different populations.

Future therapeutic directions should include developing orally bioavailable TMA lyase inhibitors with minimal off-target effects, identifying probiotic strains and prebiotic formulations that consistently lower TMAO across populations, and designing trials that integrate dietary and microbial endpoints to determine whether TMAO reduction improves disease outcomes.

## 9 Conclusions

TMAO is a complex metabolite at the intersection of diet, microbiota, and host physiology, exhibiting harmful and protective effects. Its ability to induce oxidative stress, inflammation, and mitochondrial dysfunction positions it as a contributor to a broad spectrum of chronic diseases. Simultaneously, its roles in protein stabilization and redox balance highlight its evolutionary importance in cellular protection. The impact of TMAO appears highly context-dependent, modulated by concentration, tissue-specific expression, and underlying disease states. Future research should identify thresholds for TMAO's beneficial versus detrimental actions, develop targeted interventions to modulate TMAO pathways, and explore personalized strategies based on microbiome and metabolic profiles.

## CRediT authorship contribution statement

**Erta Rajabi:** Writing – review & editing, Writing – original draft. **Zahra Pourramezan Seyedi:** Investigation, Project administration, Writing – original draft, Writing – review & editing. **Mahsa Ghamari:** Writing – review & editing, Writing – original draft. **Roghayeh Mohammadzadeh:** Writing – review & editing, Writing – original draft. **Mehdi Mansouri:** Writing – original draft, Writing – review & editing. **Friedrich Götz:** Writing – original draft, Validation, Supervision, Conceptualization. **Narjess Bostanghadiri:** Writing – review & editing, Writing – original draft, Project administration, Investigation, Data curation, Conceptualization.

## Informed consent

Not applicable.

## Organ donation

Not applicable.

## Ethical statement

Not applicable.

## Data availability statement

Data sharing does not apply to this article, as no datasets were generated during the current study.

## Animal treatment

Not applicable.

## Generative AI

No generative artificial intelligence (AI) tools were used in the preparation, writing, editing, analysis, or revision of this manuscript.

## Funding

Not applicable.

## Declaration of competing interest

The authors declare that they have no known competing financial interests or personal relationships that could have appeared to influence the work reported in this paper.
